# Supplementary material for: Empirical Dietary Patterns Associated with Food Insecurity in U.S. Cancer Survivors: NHANES 1999–2018
Source: Int J Environ Res Public Health. 2022 Oct 28;19(21):14062. doi: 10.3390/ijerph192114062 (PMC9656362; doi:10.3390/ijerph192114062)
Supplement: Supplementary file 1 [file ijerph-19-14062-s001.zip › ijerph-1974824-supplementary.pdf]

**Supplementary Table S1.** Food grouping scheme used in the present analysis.

| **Food Group** [a] | **Description** |
|---|---|
| Processed Meats | *Oz cooked lean meat from franks, sausages, luncheon meats* |
| Meats | *Oz cooked lean meat from beef, pork, veal, lamb, organ meats, and game* |
| Poultry | *Oz cooked lean meat from chicken, poultry, and other poultry* |
| Seafood—High n-3 | *Oz cooked lean meat from fish, other seafood high in Omega-3* |
| Seafood—Low n-3 | *Oz cooked lean meat from fish, other seafood low in Omega-3* |
| Eggs | *Oz equivalents of eggs* |
| Solid Fats | *Grams of discretionary Solid fat* |
| Oils | *Grams of discretionary Oil* |
| Milk | *Number of milk cup equivalents* |
| Yogurt | *Number of yogurt cup equivalents* |
| Cheese | *Number of cheese cup equivalents* |
| Alcohol | *Total drinks of alcohol* |
| Fruit—Other | *Number of other fruit cup equivalents* |
| Fruit—Citrus, melons, and berries | *Number of citrus, melon, berry cup equivalents* |
| Tomatoes | *Number of tomato cup equivalents* |
| Dark-Green Vegetables | *Number of dark-green vegetable cup equivalents* |
| Dark-Yellow Vegetables | *Number of orange vegetable cup equivalents* |
| Other Vegetables | *Number of other vegetable cup equivalents* |
| Potatoes | *Number of white potato cup equivalents* |
| Other Starchy Vegetables | *Number of other starchy vegetable cup equivalents* |
| Legumes | *Number of cooked dry beans and peas cup equivalents* |
| Soy | *Oz equivalents of soy products* |
| Refined Grains | *Number of non-whole grain ounce equivalents* |
| Whole Grains | *Number of whole grain ounce equivalents* |
| Nuts | *Oz equivalents of nuts and seeds* |
| Added Sugars | *Teaspoon equivalents of added sugars* |

[a] Adopted from the FPED/MPED Database.

**Supplementary Table S2.** Model coefficients or component loadings for each of the derived patterns (extracted using either penalized logistic regression or principal components analysis) and the food groups used in the analysis. This analysis was performed on subsample A (Figure 1), an analytical subsample of food insecure cancer survivors (*n* = 3317).

| Pattern<br>Food Groups | Food Insecurity (FI) † | Age † | Food Assistance (SNAP) † | Household Size † | Modified Western ‡ | Prudent ‡ |
|---|---|---|---|---|---|---|
| Processed Meats | 0.00 | 0.00 | 0.00 | 0.01 | 0.17 | -0.12 |
| Meats | 0.15 | -0.05 | 0.08 | 0.01 | 0.24 | -0.13 |
| Poultry | 0.11 | -0.18 | 0.05 | 0.05 | 0.14 | 0.26 |
| Seafood—High n-3 | 0.00 | 0.00 | 0.03 | 0.00 | 0.01 | 0.13 |
| Seafood—Low n-3 | 0.00 | 0.02 | 0.05 | -0.02 | 0.07 | -0.03 |
| Eggs | 0.10 | 0.03 | 0.07 | 0.04 | 0.20 | 0.05 |
| Solid Fats | 0.00 | 0.16 | -0.08 | -0.05 | 0.39 | -0.19 |
| Oils | 0.00 | 0.02 | 0.00 | 0.02 | 0.34 | -0.05 |
| Milk | 0.00 | 0.12 | 0.00 | -0.02 | 0.20 | 0.03 |
| Yogurt | 0.00 | 0.01 | 0.00 | -0.04 | 0.09 | 0.21 |
| Cheese | 0.05 | -0.25 | 0.10 | -0.02 | 0.25 | -0.18 |
| Alcohol | -0.01 | -0.09 | -0.25 | -0.01 | 0.05 | -0.20 |
| Fruit—Other | 0.00 | 0.21 | -0.06 | -0.04 | 0.12 | 0.30 |
| Fruit—Citrus, melons, and berries | 0.00 | 0.06 | 0.00 | -0.03 | 0.07 | 0.40 |
| Tomatoes | 0.00 | 0.01 | 0.00 | -0.04 | 0.27 | -0.05 |
| Dark-Green Vegetables | 0.00 | -0.17 | 0.00 | -0.01 | 0.12 | 0.33 |
| Dark-Yellow Vegetables | 0.00 | 0.00 | -0.16 | 0.01 | 0.12 | 0.32 |
| Other Vegetables | -0.19 | 0.11 | -0.49 | -0.06 | 0.22 | 0.27 |
| Potatoes | 0.21 | 0.14 | 0.00 | 0.00 | 0.23 | 0.01 |
| Other Starchy Vegetables | 0.00 | 0.10 | -0.08 | -0.03 | 0.03 | 0.17 |
| Legumes | 0.06 | -0.19 | 0.25 | 0.05 | 0.11 | -0.15 |
| Soy | 0.00 | -0.05 | -0.12 | 0.04 | 0.12 | 0.10 |
| Refined Grains | 0.00 | 0.00 | 0.00 | 0.02 | 0.34 | -0.16 |
| Whole Grains | 0.00 | 0.26 | -0.15 | -0.04 | 0.14 | 0.26 |

| | | | | | | |
|---|---|---|---|---|---|---|
| Nuts | -0.03 | 0.05 | -0.17 | 0.00 | 0.16 | -0.01 |
| Added Sugars | 0.41 | -0.17 | 0.38 | 0.07 | 0.24 | -0.18 |

† Dietary pattern obtained using penalized logistic regression.

‡ Dietary patterns obtained using principal components analysis (PCA).

**Supplementary Table S3.** Stratified odds ratios[†] and 95% confidence intervals for the relationship between the dietary patterns scores and the odds of being food insecure.

| Dietary Pattern [a] | Group | Q1 | Q2 | Q3 | Q4 | Q5 | $p_{Q5\text{-}Q1}$ | $p_{trend}$ | OR [b] continuous | $p$ [c] quadratic |
|---|---|---|---|---|---|---|---|---|---|---|
| Food Insecurity [†] | Male | 1.00 | 0.65 (0.20-2.11) | 0.95 (0.34-2.66) | 1.87 (0.70-4.99) | 1.46 (0.44-4.87) | 0.53 | 0.23 | 1.32 (0.89-1.96) | 0.23 |
| Age [†] | Male | 1.00 | 1.04 (0.34-3.15) | 1.52 (0.51-4.56) | 1.37 (0.33-5.60) | 2.68 (0.82-8.72) | 0.10 | 0.13 | 1.31 (0.95-1.81) | 0.96 |
| Food Assistance (SNAP) [†] | Male | 1.00 | 0.48 (0.15-1.53) | 0.56 (0.22-1.42) | 0.82 (0.29-2.34) | 1.09 (0.33-3.59) | 0.88 | 0.59 | 1.17 (0.77-1.78) | 0.84 |
| Household Size [†] | Male | 1.00 | 0.76 (0.26-2.26) | 0.51 (0.14-1.82) | 0.99 (0.29-3.30) | 0.97 (0.37-2.56) | 0.96 | 0.68 | 0.94 (0.71-1.23) | 0.19 |
| Modified Western [‡] | Male | 1.00 | 1.90 (0.68-5.29) | 0.67 (0.23-1.98) | 1.21 (0.46-3.19) | 0.30 (0.11-0.85)* | 0.02* | 0.01* | 0.75 (0.58-0.96)* | 0.47 |
| Prudent [‡] | Male | 1.00 | 2.30 (0.79-6.72) | 2.38 (1.04-5.46)* | 2.00 (0.71-5.64) | 1.82 (0.69-4.81) | 0.23 | 0.35 | 1.17 (0.79-1.74) | 0.10 |
| Food Insecurity [†] | Female | 1.00 | 1.65 (0.76-3.59) | 1.82 (0.71-4.64) | 2.26 (1.00-5.10) | 3.48 (1.49-8.09)** | <0.01** | <0.01** | 1.59 (1.22-2.07)** | 0.14 |
| Age [†] | Female | 1.00 | 1.80 (1.00-3.26) | 1.39 (0.61-3.19) | 1.02 (0.37-2.83) | 1.37 (0.67-2.82) | 0.38 | 0.81 | 0.97 (0.75-1.25) | 0.19 |
| Food Assistance (SNAP) [†] | Female | 1.00 | 2.09 (0.84-5.18) | 2.11 (1.04-4.28)* | 3.80 (1.73-8.33)** | 3.20 (1.63-6.28)** | <0.01** | <0.01** | 1.53 (1.21-1.93)** | 0.33 |
| Household Size [†] | Female | 1.00 | 1.66 (0.70-3.95) | 1.45 (0.70-3.01) | 3.74 (1.85-7.54)** | 2.71 (1.16-6.35)* | 0.02* | <0.01** | 1.45 (1.13-1.86)** | 0.48 |
| Modified Western [‡] | Female | 1.00 | 0.36 (0.16-0.79)* | 0.77 (0.37-1.59) | 1.55 (0.81-2.95) | 1.13 (0.52-2.47) | 0.76 | 0.12 | 1.14 (0.92-1.41) | 0.87 |
| Prudent [‡] | Female | 1.00 | 0.72 (0.27-1.91) | 1.24 (0.52-2.96) | 0.41 (0.16-1.02) | 0.31 (0.14-0.69)** | <0.01** | <0.01** | 0.68 (0.54-0.86)** | 0.27 |
| Food Insecurity [†] | 2-6 Years Since Dx | 1.00 | 3.11 (0.70-13.80) | 1.41 (0.32-6.28) | 2.14 (0.48-9.62) | 4.72 (1.26-17.70)* | 0.02* | 0.02* | 1.39 (1.03-1.88)* | 0.85 |
| Age [†] | 2-6 Years Since Dx | 1.00 | 1.03 (0.34-3.11) | 0.67 (0.19-2.29) | 1.33 (0.34-5.19) | 0.54 (0.14-2.08) | 0.37 | 0.54 | 0.79 (0.57-1.1) | 0.67 |
| Food Assistance (SNAP) [†] | 2-6 Years Since Dx | 1.00 | 3.78 (0.66-21.76) | 5.27 (0.96-28.93) | 6.42 (1.30-31.81)* | 7.90 (2.18-28.66)** | <0.01** | <0.01** | 1.65 (1.16-2.34)** | 0.35 |
| Household Size [†] | 2-6 Years Since Dx | 1.00 | 7.66 (1.75-33.53)** | 2.45 (0.52-11.56) | 4.22 (0.84-21.19) | 5.56 (1.37-22.55)* | 0.02* | 0.16 | 1.30 (0.92-1.82) | 0.72 |
| Modified Western [‡] | 2-6 Years Since Dx | 1.00 | 1.10 (0.27-4.52) | 0.79 (0.23-2.74) | 4.98 (1.73-14.37)** | 1.32 (0.41-4.28) | 0.64 | 0.13 | 1.23 (0.86-1.74) | 0.43 |
| Prudent [‡] | 2-6 Years Since Dx | 1.00 | 0.94 (0.31-2.84) | 1.27 (0.41-3.97) | 0.86 (0.26-2.89) | 0.50 (0.12-2.11) | 0.35 | 0.36 | 0.80 (0.51-1.25) | 0.17 |
| Food Insecurity [†] | ≥6 Years Since Dx | 1.00 | 0.69 (0.33-1.45) | 1.02 (0.43-2.42) | 1.54 (0.78-3.04) | 2.11 (0.86-5.13) | 0.10 | 0.03* | 1.54 (1.13-2.12)** | 0.14 |
| Age [†] | ≥6 Years Since Dx | 1.00 | 2.66 (1.41-5.04)** | 1.42 (0.57-3.56) | 0.99 (0.30-3.28) | 2.79 (1.23-6.32)* | 0.01* | 0.25 | 1.14 (0.89-1.47) | 0.53 |
| Food Assistance (SNAP) [†] | ≥6 Years Since Dx | 1.00 | 1.15 (0.46-2.88) | 0.98 (0.45-2.13) | 2.35 (0.88-6.24) | 1.94 (0.80-4.69) | 0.14 | 0.08 | 1.37 (1.03-1.83)* | 0.50 |
| Household Size [†] | ≥6 Years Since Dx | 1.00 | 1.10 (0.42-2.89) | 1.01 (0.44-2.34) | 2.47 (1.16-5.26)* | 2.03 (0.79-5.21) | 0.14 | 0.02* | 1.30 (0.99-1.70) | 0.25 |
| Modified Western [‡] | ≥6 Years Since Dx | 1.00 | 0.75 (0.37-1.50) | 0.43 (0.20-0.94)* | 1.74 (0.87-3.49) | 0.90 (0.39-2.07) | 0.80 | 0.54 | 1.03 (0.82-1.28) | 0.81 |
| Prudent [‡] | ≥6 Years Since Dx | 1.00 | 0.73 (0.27-1.97) | 0.84 (0.35-2.03) | 0.39 (0.16-0.95)* | 0.33 (0.14-0.79)* | 0.01* | <0.01** | 0.64 (0.50-0.82)** | 0.04* |
| Food Insecurity [†] | ≤2 Years Since Dx | 1.00 | 2.52 (0.45-14.10) | 1.76 (0.32-9.62) | 4.01 (1.25-12.83)* | 1.74 (0.28-10.65) | 0.54 | 0.52 | 1.58 (0.93-2.69) | 0.27 |
| Age [†] | ≤2 Years Since Dx | 1.00 | 1.17 (0.19-7.35) | 2.76 (0.54-14.08) | 2.13 (0.46-9.76) | 1.83 (0.3-11.05) | 0.50 | 0.28 | 1.17 (0.73-1.88) | 0.90 |
| Food Assistance (SNAP) [†] | ≤2 Years Since Dx | 1.00 | 1.17 (0.13-10.46) | 3.00 (0.80-11.28) | 1.92 (0.60-6.16) | 0.89 (0.15-5.13) | 0.89 | 0.85 | 1.17 (0.72-1.88) | 0.72 |
| Household Size [†] | ≤2 Years Since Dx | 1.00 | 1.83 (0.46-7.24) | 1.33 (0.26-6.73) | 3.06 (0.78-12) | 1.87 (0.5-6.97) | 0.34 | 0.35 | 1.04 (0.67-1.62) | 0.29 |

| | | | | | | | | | | |
|---|---|---|---|---|---|---|---|---|---|---|
| Modified Western ‡ | ≤2 Years Since Dx | 1.00 | 1.69 (0.22-12.76) | 2.08 (0.42-10.38) | 1.64 (0.39-6.89) | 1.80 (0.37-8.83) | 0.46 | 0.48 | 0.98 (0.63-1.53) | 0.49 |
| Pruden t‡ | ≤2 Years Since Dx | 1.00 | 2.86 (0.57-14.26) | 4.59 (0.92-23) | 2.18 (0.45-10.58) | 1.02 (0.22-4.65) | 0.98 | 0.74 | 1.11 (0.81-1.52) | 0.56 |
| Food Insecurity † | ≤ High School | 1.00 | 0.55 (0.26-1.16) | 0.81 (0.35-1.87) | 1.20 (0.56-2.57) | 0.91 (0.43-1.91) | 0.79 | 0.69 | 1.15 (0.9-1.48) | 0.16 |
| Age † | ≤ High School | 1.00 | 2.12 (1.03-4.36)* | 1.42 (0.54-3.74) | 1.15 (0.46-2.87) | 1.39 (0.61-3.18) | 0.43 | 0.72 | 0.96 (0.74-1.25) | 0.24 |
| Food Assistance (SNAP) † | ≤ High School | 1.00 | 0.88 (0.36-2.11) | 1.16 (0.58-2.33) | 1.58 (0.75-3.30) | 1.11 (0.52-2.38) | 0.78 | 0.45 | 1.08 (0.84-1.39) | 0.69 |
| Household Size † | ≤ High School | 1.00 | 1.81 (0.82-3.97) | 0.92 (0.39-2.16) | 1.94 (0.78-4.83) | 1.39 (0.58-3.33) | 0.46 | 0.47 | 1.06 (0.83-1.36) | 0.93 |
| Modified Western ‡ | ≤ High School | 1.00 | 0.81 (0.33-1.99) | 0.77 (0.30-1.94) | 1.01 (0.43-2.39) | 1.10 (0.5-2.43) | 0.81 | 0.72 | 1.01 (0.76-1.36) | 0.18 |
| Prudent ‡ | ≤ High School | 1.00 | 1.14 (0.59-2.21) | 1.33 (0.58-3.08) | 0.76 (0.32-1.84) | 0.76 (0.34-1.67) | 0.49 | 0.35 | 0.98 (0.76-1.26) | 0.75 |
| Food Insecurity † | ≥ Some College | 1.00 | 2.34 (0.83-6.59) | 2.98 (1.00-8.88)* | 3.30 (0.98-11.06) | 9.21 (3.37-25.13)** | <0.01** | <0.01** | 2.12 (1.46-3.07)** | 0.14 |
| Age † | ≥ Some College | 1.00 | 1.36 (0.61-3.03) | 0.81 (0.25-2.59) | 1.64 (0.41-6.57) | 1.89 (0.69-5.12) | 0.21 | 0.27 | 1.11 (0.79-1.55) | 0.84 |
| Food Assistance (SNAP) † | ≥ Some College | 1.00 | 3.64 (1.33-9.97)* | 3.06 (1.32-7.10)** | 5.76 (1.87-17.71)** | 6.07 (2.25-16.37)** | <0.01** | <0.01** | 1.88 (1.33-2.65)** | 0.44 |
| Household Size † | ≥ Some College | 1.00 | 1.44 (0.39-5.37) | 0.81 (0.31-2.14) | 2.50 (0.85-7.35) | 4.68 (1.78-12.27)** | <0.01** | <0.01** | 1.8 (1.32-2.45)** | 0.66 |
| Modified Western ‡ | ≥ Some College | 1.00 | 0.66 (0.24-1.79) | 0.76 (0.29-1.99) | 2.21 (0.98-4.98) | 0.9 (0.32-2.53) | 0.84 | 0.42 | 1.08 (0.82-1.41) | 0.40 |
| Prudent ‡ | ≥ Some College | 1.00 | 0.67 (0.17-2.61) | 1.02 (0.32-3.24) | 0.52 (0.18-1.47) | 0.23 (0.07-0.73)* | 0.01* | 0.01* | 0.57 (0.42-0.78)** | 0.02* |

** $p < 0.01$, * $p < 0.05$. [a] All models adjusted for age, sex, race/ethnicity, family income-to-poverty ratio, highest level of education attained, household size, SNAP participation status, BMI, estimated caloric intake, weekly MET minutes, primary cancer site, smoking status, and the Charlson Comorbidity Index score and were weighted according to guidelines provided by the NCHS. [b] Odds ratio (HR) corresponding to a standard deviation increase in the diet pattern score. [c] Wald test *p*-value for a quadratic polynomial term. † Dietary pattern obtained using penalized logistic regression. ‡ Dietary pattern obtained using principal components analysis (PCA).

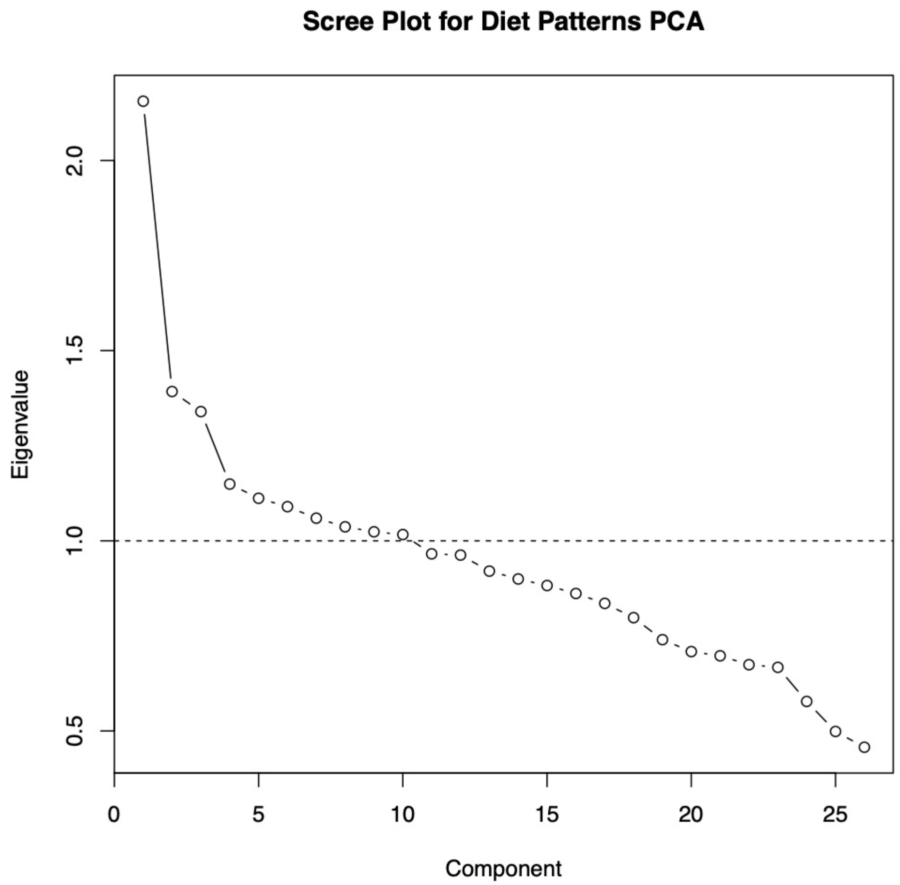


**Supplementary Figure S1.** Scree plot from the PCA dietary extractions procedure performed on subsample B *(n* = 433).
